# FDG uptake tracks the oxidative damage in diabetic skeletal muscle: An experimental study

**DOI:** 10.1016/j.molmet.2019.11.007

**Published:** 2019-11-15

**Authors:** Matteo Bauckneht, Vanessa Cossu, Patrizia Castellani, Patrizia Piccioli, Anna Maria Orengo, Laura Emionite, Francesco Di Giulio, Maria Isabella Donegani, Alberto Miceli, Stefano Raffa, Anna Borra, Selene Capitanio, Silvia Morbelli, Giacomo Caviglia, Silvia Bruno, Silvia Ravera, Davide Maggi, Gianmario Sambuceti, Cecilia Marini

**Affiliations:** 1 Nuclear Medicine, IRCCS Ospedale Policlinico San Martino, Largo Benzi 10, 16132 Genoa, Italy; 2 Department of Health Sciences, University of Genoa, Via Pastore 1, 16132 Genoa, Italy; 3 Cell Biology Unit, IRCCS Ospedale Policlinico San Martino, Largo Benzi 10, 16132 Genoa, Italy; 4 Animal Facility, IRCCS Ospedale Policlinico San Martino, Largo Benzi 10, 16132 Genoa, Italy; 5 Department Experimental Medicine, University of Genoa, Len Battista Alberti 2, 16132 Genoa, Italy; 6 Department of Internal Medicine, University of Genoa, Viale Benedetto XV 6, 16132 Genoa, Italy; 7 Diabetes Unit, IRCCS Ospedale Policlinico San Martino Genoa, Largo Benzi 10, 16132 Genoa, Italy; 8 Department of Mathematics (DIMA), University of Genoa, Via Dodecaneso 35, 16146 Genoa, Italy; 9 CNR Institute of Molecular Bioimaging and Physiology (IBFM), Via Fratelli Cervi 93, 20090 Segrate (MI), Italy

**Keywords:** Fluorodeoxyglucose, Hexose-6P-dehydrogenase, Diabetes, Skeletal muscle, Oxidative stress

## Abstract

**Objectives:**

The present study aims to verify the relationship between glucose consumption and uptake of ^18^F-2-deoxy-glucose (FDG) in the skeletal muscle (SM) of experimental models of streptozotocin-induced diabetes mellitus (STZ-DM).

**Methods:**

The study included 36 Balb/c mice. Two weeks after intraperitoneal administration of saline (control group, n = 18) or 150 mg streptozotocin (STZ-DM group, n = 18), the two cohorts were submitted to an oral glucose tolerance test and were further subdivided into three groups (n = 6 each): untreated and treated with metformin (MTF) at low or high doses (10 or 750 mg/kg daily, respectively). Two weeks thereafter, all mice were submitted to dynamic micro–positron emission tomography (PET) imaging after prolonged fasting. After sacrifice, enzymatic pathways and response to oxidative stress were evaluated in harvested SM.

**Results:**

On PET imaging, the FDG uptake rate in hindlimb SM was significantly lower in nondiabetic mice as compared with STZ-DM–untreated mice. MTF had no significant effect on SM FDG uptake in untreated mice; however, its high dose induced a significant decrease in STZ-DM animals. Upon conventional analysis, the SM standard uptake value was higher in STZ-DM mice, while MTF was virtually ineffective in either control or STZ-DM models. This metabolic reprogramming was not explained by any change in cytosolic glucose metabolism. By contrast, it closely agreed with the catalytic function of hexose-6P-dehydrogenase (H6PD; i.e., the trigger of a specific pentose phosphate pathway selectively located within the endoplasmic reticulum). In agreement with this role, the H6PD enzymatic response to both STZ-DM and MTF matched the activation of the NADPH-dependent antioxidant responses to the increased generation of reactive oxygen species caused by chronic hyperglycemia. Ex vivo analysis of tracer kinetics confirmed that the enhanced SM avidity for FDG occurred despite a significant reduction in glucose consumption, while it was associated with increased radioactivity transfer to the endoplasmic reticulum.

**Conclusions:**

These data challenge the current dogma linking FDG uptake to the glycolytic rate. They instead introduce a new model considering a strict link between the uptake of this glucose analog, H6PD reticular activity, and oxidative damage in diabetes, at least under fasting condition.

## Highlights

• The accepted link between glucose consumption and uptake of hexose analogs such as FDG and 2DG is looser than expected.
• FDG selectively tracks the activity of a pentose-phosphate shunt triggered by H6PD.
• FDG uptake in the skeletal muscles tracks the hyperglycemia-related oxidative damage.
• FDG uptake might represent an index of NADPH renewal indicating the ER-PPP response to oxidative stress.

## 1 Introduction

Skeletal muscle (SM) is a key player in the pathogenesis of type 2 diabetes mellitus, accounting for up to 85% of insulin's effect on blood clearance of glucose under physiologic conditions [1,2]. According to this, a substantial body of literature has focused on the mechanisms regulating SM glucose intake as a target of antihyperglycemic therapies [2,3]. Until the late 1970s, this issue had been most often investigated using leg glucose metabolism with balance techniques and chemical tissue analysis [4]. However, in the past decades, this complex methodology has been replaced by imaging techniques and, in particular, by the analysis of ^18^F-fluoro-deoxyglucose (FDG) kinetics with positron emission tomography (PET), which so far represents a reference method for studying muscular glucose metabolism (MRGlu).

The theoretical basis for this approach has been described by Phelps et al. [5], who extended FDG to the kinetic model previously described by Sokoloff and coworkers for brain uptake of ^14^C-2-deoxyglucose [6]. According to this model, FDG competes with glucose for transmembrane transport and hexokinase (HK)–catalyzed entrapment mechanism. However, once phosphorylated to FDG-6P, it is irreversibly trapped in the tissue, making late tissue radioactivity content a robust index of overall glucose intake.

One of the major advantages offered by FDG-PET imaging is the possibility to simultaneously evaluate tracer time–concentration curves in arterial blood and interrogated tissue and thus to apply compartmental analysis to characterize the effect of insulin on SM glucose kinetics. Nevertheless, this approach surprisingly reported only a trivial response of transmembrane transport (usually termed as k_1_) to insulin that instead caused a 6- to 10-fold increase in the hexose phosphorylation rate (k_3_) both in insulin-sensitive [1] and insulin-resistant subjects [2]. This response profoundly disagrees not only with the expected hormone effect on GLUT-4 membrane docking [7] but also with the documented behavior of either ^13^C-labeled glucose [8] or the inert hexose [O-Methyl-^11^C]3-O-Methyl-d-Glucose [3] not accessible to HK, which showed a marked increase in transmembrane transport under insulin stimulation.

This recognized disagreement [9] suggests that MRGlu and FDG uptake might reflect at least partially different mechanisms. This concept agrees with previous evidence in the brain [10,11], myocardium [12], and cancer [14] in which FDG uptake was found to be largely independent of glycolytic flux and strictly linked to the activity of hexose-6-phosphate dehydrogenase (H6PD). This autosomic counterpart of glucose-6P-dehydrogenase (G6PD) recognizes a large number of phosphorylated and free hexoses to catalyze the first two reactions of a pentose phosphate pathway (PPP hexose-6-phosphate dehydrogenase), selectively confined inside the endoplasmic reticulum (ER) [14].

Hyperglycemia-related oxidative load significantly contributes to the damage [15] to SM structure and function caused by diabetes [16]. The extension of redox stress to the ER environment might thus activate the local H6PD-triggered PPP to fuel the NADPH levels needed for the antioxidant response [[10], [11], [12], [13], [14], [15], [16], [17], [18], [19]].

To verify this hypothesis, we evaluated FDG kinetics in the SM of experimental models of type 2 diabetes induced by streptozotocin (STZ-DM). Moreover, to verify the link between tracer uptake and reactive oxygen species (ROS) generation, we also tested the metabolic response to the respiratory inhibition caused by metformin (MTF) treatment, since this drug is widely used in type 2 DM patients and can reduce FDG uptake despite an accelerated glycolytic flux in experimental cancers [[20], [21], [22], [23], [24], [25], [26], [27], [28]].

## 2 Material and methods

### 2.1 Chemicals

MTF and STZ were provided by Sigma–Aldrich (St. Louis, USA). FDG was produced according to standard methodology. Glucose and 2DG were provided by Sigma–Aldrich (St. Louis, USA). Ketamine and xylazine were provide by Imalgene (Milan, Italy) and Bio98 (Italy).

### 2.2 In vivo study

#### 2.2.1 Animal models

All animal experiments were performed in accordance with guidelines and regulations (Italian 26/2014 and EU 2010/63/UE directives) after approval of both the local Ethical Committee and Italian Ministry of Health (Ministry authorization No. 832/2016/PR). The study included 36 six-week-old male BALB/c mice (Charles River, Italy): 18 in the STZ-DM group, which received intraperitoneal STZ (150 mg), and 18 controls that received saline. Two weeks thereafter, an oral glucose tolerance test (OGTT) was performed by administering a glucose load (1 g/kg) and assaying serum glucose level at 15, 30, 60, and 120 min. The day after, both cohorts were divided into three groups (n = 6 per cohort) that were untreated or treated with MTF diluted in drinking water to administer a daily dose of 10 (MTF-10) or 750 mg/kg (MTF-750) (n = 6 per dose), respectively. Treatment was continued for two weeks until the day of micro-PET imaging. Time features of the study protocol are depicted in Supplementary Figure 1.

#### 2.2.2 Experimental micro-PET imaging

Two weeks after OGTT, mice were fasted for 12 h, weighed, and anesthetized with intraperitoneal ketamine (100 mg/kg) and xylazine (10 mg/kg). Serum glucose level was measured, and animals were positioned in a dedicated micro-PET system (Albira, Bruker, USA). FDG (3–4 MBq) was injected through a tail vein, soon after the start of a 50-minute list-mode acquisition. The day after imaging, mice were euthanized by cervical dislocation, and SMs were harvested from 3 mice per group and submitted to immunohistochemical and biochemical analysis.

#### 2.2.3 Image processing

According to our procedure [[10], [11], [12], [13],^18^,^27^], acquisition was binned in the following frames: 10 × 15 s, 5 × 30 s, 2 × 150 s, 6 × 300 s, and 1 × 600 s; images were reconstructed using the maximal likelihood expectation maximization method. An experienced observer identified a volume of interest (VOI) in the left ventricle to plot the arterial input function that served for FDG clearance calculation. Parametric maps of the FDG accumulation rate were thus obtained according to the Gjedde-Patlak graphical approach [29] using the routine of commercial software (PMOD, Zurich, CH). A VOI was drawn on leg SMs to estimate the average FDG avidity expressed by the slope of the Patlak regression line ($\frac{k_{1}\times k_{3}}{k_{2}+k_{3}},min^{-1}$), which was also multiplied for serum glucose level to estimate the average MRGlu, in nMol × min^−1^ ×g^−1^. The same VOI was copied on the last frame of the original acquisition to estimate the FDG standard uptake value (SUV).

### 2.3 Ex vivo study

#### 2.3.1 Ex vivo evaluation of glucose and FDG uptake

Ex vivo SM uptake of glucose and FDG was evaluated in the quadriceps muscles of the three control and STZ-DM mice soon after sacrifice using the LigandTracer white instrument (Ridgeview, Uppsala, Sweden) according to our previously validated procedure [10]. The side of the studied quadriceps was randomly selected. Samples of MTF-treated mice were not studied considering the impossibility to reproduce the in vivo drug concentration. Briefly, 300-μm-thick SM slices were stuck in the outer ring of a Petri dish with octyl-cyanoacrylate (Dermabond, Ethicon, US) and covered with 3 mL solution collected from a 5-mL vial of Dulbecco's Modified Eagle's Medium containing glucose and FDG at the concentration of 5.5 mM (1 g/L) and 2 MBq/mL, respectively. The Petri dish was placed on the plate of the instrument, whose inclination at 30° from the horizon permits obtaining the time–activity curve of tissue FDG uptake, while switching the sample position from the system nadir (for incubation) to zenith (for counting) every 30 s. After 45 min, an aliquot of 0.5 mL was sampled both from the input vial and from the Petri dish (output) to measure the radioactivity concentration using a dose calibrator with an activity resolution <10 KBq (Capintec CRC55). Fractional FDG uptake was thus calculated as

$$\text{Fractional FDG uptake}=\frac{{\text{A}}_{\text{input}}-{\text{A}}_{\text{output}}}{{\text{A}}_{\text{input}}}$$

where A_input_ and A_output_ represent activity (MBq) in the input and the output, respectively.

Glucose consumption was measured as

Glucose intake = [C_input_−C_output_] × 3 × 10^−3^

where C_input_ and C_output_ represent the corresponding mM concentrations, and 3×10^−3^ represents the volume of used medium.

FDG time–activity curves were analyzed using our compartmental-analysis approach previously validated in cancer cell cultures [30] based on the assumption that FDG-6P can be processed only by H6PD or hydrolyzed by G6P-phosphatase (G6Pase) and thus in the ER lumen where both enzymes are confined. The proposed model thus considers the conventional rate constants describing FDG bidirectional transport through sarcolemma (conventionally defined as k_1_ and k_2_, respectively) and HK-catalyzed phosphorylation (k_3_). However, it also considers FDG6P transport though the ER membrane (defined as k_5_) as the needed step for tracer access to G6Pase-catalyzed hydrolysis and back-diffusion to the cytosol (defined as k_6_) facilitated by the high abundance of GLUTs in ER membrane. The inverse problem of compartmental analysis was solved by means of a Newton-type iterative algorithm, already used and validated by our group [[31], [32], [33], [34], [35]].

#### 2.3.2 Spectrophotometric assay

Soon after sacrifice, one quadriceps muscle was harvested from each mouse by randomly selecting the side. These samples were homogenized in phosphate buffered saline (PBS) supplemented by protease inhibitors with a Potter-Elvehjem homogenizer, and enzymatic assays were performed as previously described [10,11].

Enzymatic assays were performed in a double-beam spectrophotometer (UNICAM UV2, Analytical S.n.c., Italy) [13].

HK, H6PD, and G6PD activities were assayed following the NADP reduction at 340 nm. Phosphofructokinase (PFK) activity was assayed following the NADH oxidation at 340 nm. The following assays solutions were used: 1) HK: 100 mM Tris–HCl pH 7.4 (TRIS7.4), 2 mM MgCl_2_, 200 mM glucose, 1 mM ATP, 0.5 mM NADP, 4 μg G6PD (Sigma–Aldrich); 2) H6PD: 100 mM TRIS7.4, 10 mM 2-deoxy-glucose-6 phosphate (2DG6P), 0.5 mM NADP mM; G6PD: 100 mM TRIS7.4, 10 mM glucose-6 phosphate (G6P), 0.5 mM NADP; 3) PFK: 100 mM Tris–HCl pH 8, 2 mM MgCl_2_, 5 mM KCl, 2 mM fructose-6-phosphate, 1 mM ATP, 0.5 mM phosphoenolpyruvate (PEP), 0.2 mM NADH, 4 μg pyruvate kinase (PK)/lactate dehydrogenase (LDH) (Sigma–Aldrich). Glutathione reductase (GR) activity and the NADPH/NADP ratio in SM homogenates were evaluated spectrophotometrically, at 405 and 450 nm, respectively, using a GR assay kit (Abcam: ab83461) and NADP-NADPH assay kit (Abcam: ab65349), following the manufacturer's instructions. Glucose in the medium was assayed following the reduction of NADP at 340 nm. The following solutions were used: glucose: 100 mM TRIS7.4, 2 mM MgCl_2_, 2 mM ATP, 4 μg HK/G6PD (Sigma–Aldrich).

G6Pase activity on G6P was measured at 660 nm, following the inorganic phosphate production [36], using 100 μg SM proteins. The assay solution contained 60 mM Tris-maleic acid pH 6.5 and 40 mM G6P. Following an incubation at 37 °C for 15 min, the reaction was blocked with 2% trichloroacetic. Samples were centrifuged at 14,000 rpm for 2 min, and the supernatant was added with 350 mM H_2_SO_4_, 350 mM ammonium molybdate, and 10 μl of 2.5 mg/ml Fiske's reactive (710 μl of final volume).

#### 2.3.3 Immunohistochemical analysis

Soon after sacrifice, the remaining quadriceps SMs of three mice per group were removed and snap frozen in OCT. Serial cryostat sections were obtained. Immediately after cutting, at least three sections were stained with the sulfhydryl-reactive dye Mercury Orange (MO; Sigma–Aldrich) as previously described [12]. Briefly, sections were incubated on ice in MO in cold 90% acetone at a final concentration of 25 μM. To confirm the specificity of MO binding to nonprotein thiols (including glutathione and cysteine), control sections were pretreated with 100 mM N-ethylmaleimide (Sigma–Aldrich) for 10 min to block thiol groups. For ROS staining, a corresponding number of sections from the same SM samples were fixed for 10 min in acetone, followed by incubation with 10 μM 2′,7′-dichlorofluorescein diacetate (H_2_DCFDA; Molecular Probes) for 30 min at 37 °C and a wash in PBS. Finally, confocal microscopy images were acquired by the Fluoview FV500 software.

### 2.4 Statistical analysis

The data are presented as mean ± standard deviation. The comparison between different groups was performed by analysis of variance for multiple comparisons; Student *t* test for paired or unpaired data were used as appropriate. Statistical significance was considered as *p* < 0.05. Analyses of differences between groups of only three samples were performed using nonparametric tests. In particular, the effect of the two MTF doses in either control or STZ-DM groups was evaluated using the Kruskal–Wallis test. On the other hand, head-to-head comparisons between control and STZ-DM groups were performed using the Mann–Whitney test. Statistical analyses were performed using PRISM software 7.0 (San Diego Ca).

## 3 Results

### 3.1 Effect of diabetes and MTF treatment on serum glucose level

**Figure 1:**
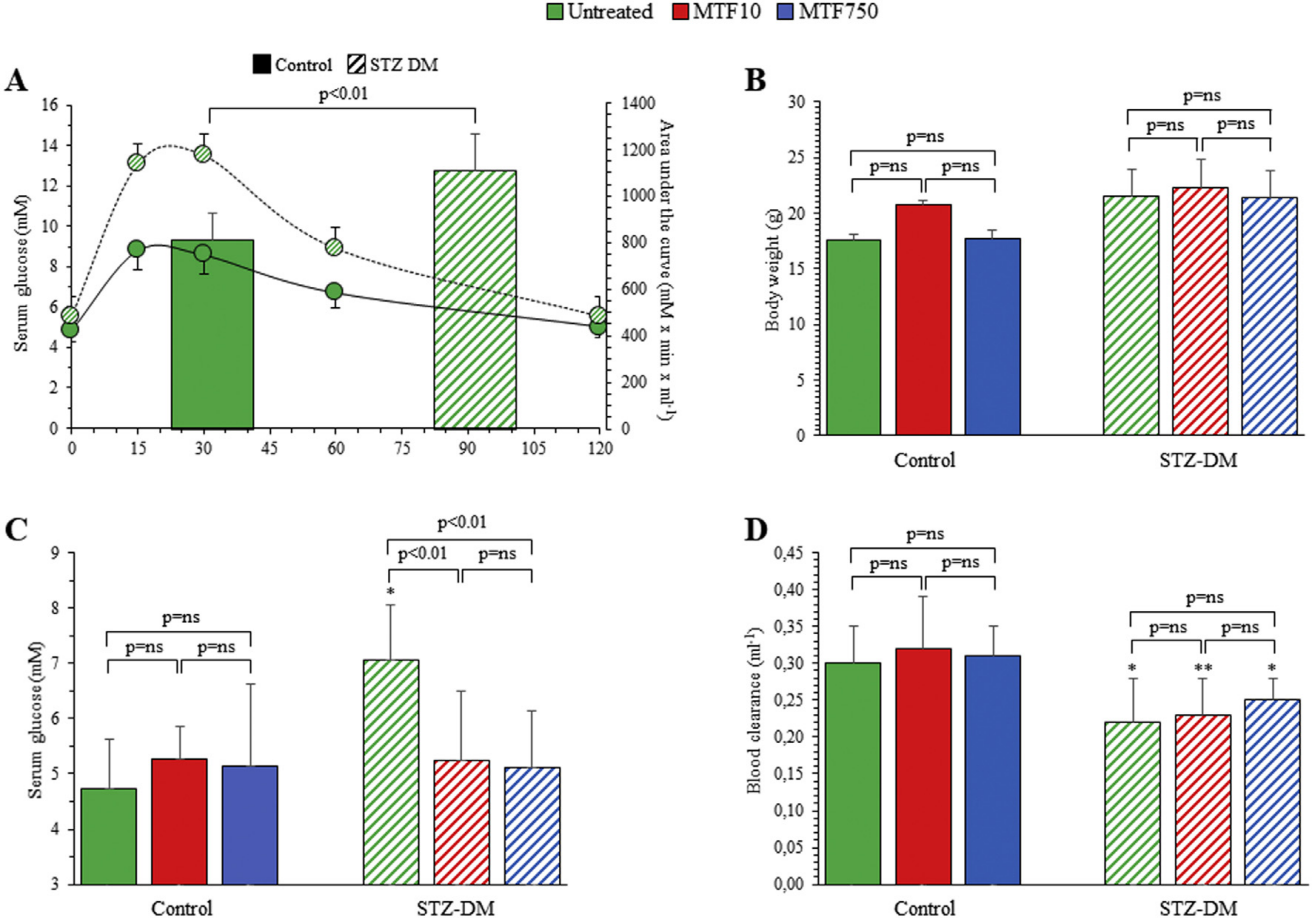
*Effect of STZ and MTF on mice serum glucose level, body weight, and clearance.* Serum glucose levels after OGTT in the 18 control (solid line) and in the 18 STZ-DM mice (dashed line) (A) (n = 18). Superimposed on this graph, with the *y*-axis on the right, is the area under the curve, with solid and dashed columns reporting average values for control and STZ-DM mice, respectively (A). (B) Body weight in control (solid columns) and STZ-DM groups (dashed columns) of mice untreated (green) or under low-dose (red) or high-dose (blue) MTF (B). Average fasting glycemia (C), measured 2 weeks thereafter and soon before PET imaging, in control (solid columns) and STZ-DM groups (dashed columns) untreated (green) and treated with either low-dose (red) or high-dose (blue) MTF (each group includes 6 mice). Blood FDG clearance (D) in control (solid columns) and STZ-DM mice (dashed columns) untreated (green) or under low- (red) or high-dose (blue) MTF (n = 6). *p < 0.05; **p < 0.01 vs the corresponding group of nondiabetic mice.

The studies were completed in all mice, and no visible side effects occurred after STZ or MTF administration. After the OGTT, the maximum glucose concentration and area under the curve were higher in STZ-DM mice than in controls (Figure 1A). Soon before PET imaging, 2 weeks after OGTT, body weight was similar in all groups (Figure 1B). At this same time, fasting glycemia became higher in STZ-untreated mice with respect to untreated controls (Figure 1C), confirming the previously described time course of response to this STZ dosage [37]. By contrast, both MTF regimens decreased serum glucose levels in STZ-DM mice to values not significantly different from those of control mice, in which the biguanide was virtually ineffective (Figure 1C). This difference matched the response of blood FDG clearance: tracer removal from the bloodstream was indeed reduced in all STZ-DM groups but not by MTF (Figure 1D).

### 3.2 Hyperglycemia and MTF effect on SM and FDG uptake

**Figure 2:**
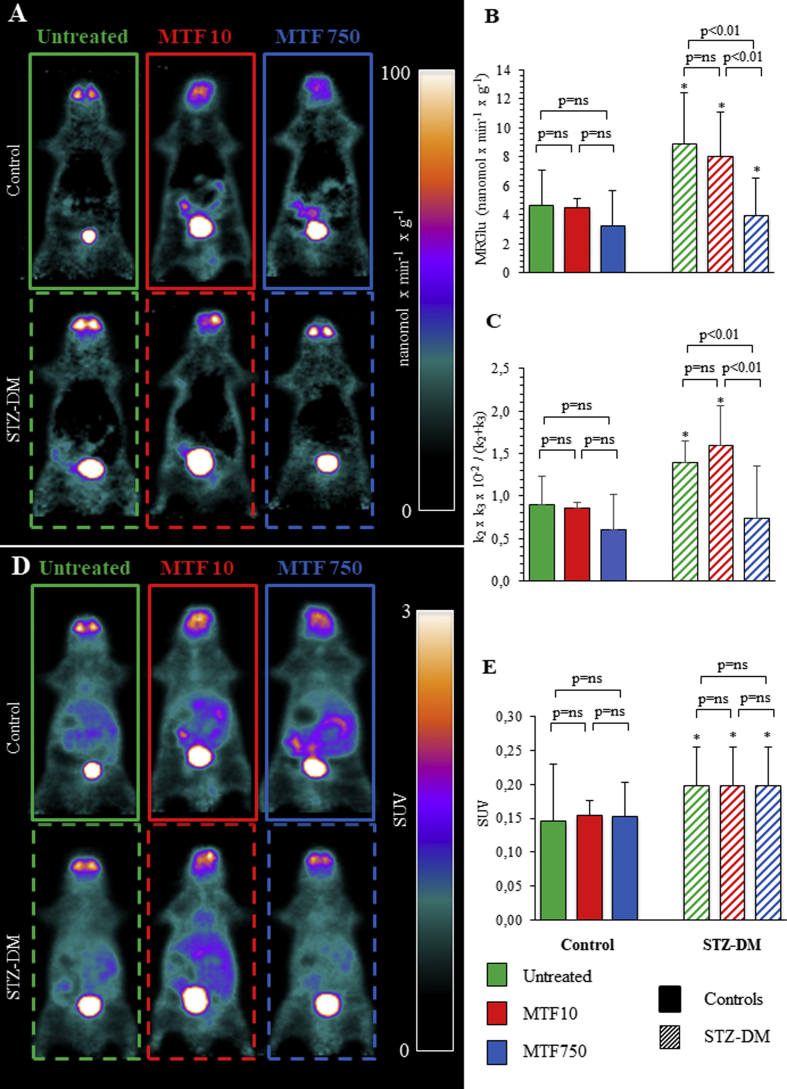
*In vivo effects of STZ-DM and metformin on SM MRGlu, slope of Patlak and SUV.* Parametric maps of representative control mice (solid line) or STZ-DM mice (dashed line), untreated (green) or under low (red, MTF10) or high (blue, MTF750) doses of MTF (A). Average glucose consumptions in control (solid columns) and STZ-DM groups (dashed columns) of mice untreated (green) or under low-dose (red) or high-dose (blue) MTF treatment (right) (B). Slope of the Patlak regression line $\frac{k_{1}\times k_{3}}{k_{2}+k_{3}}$ is expressed in panel C. SM SUV of representative mice (D) and average SUV (E). n = 6; *p < 0.05 vs the corresponding group of nondiabetic mice.

In untreated mice, FDG estimation of SM MRGlu was significantly higher in STZ-DM than in control mice (Figure 2A, B). MTF scarcely affected muscle MRGlu in nondiabetic mice, while it induced a dose-dependent decrease that became significant at the highest dose in the STZ-DM group (Figure 2A, B). This difference was only partially related to the contribution of serum glucose level, since the slope of the Patlak regression line ($\frac{k_{1}\times k_{3}}{k_{2}+k_{3}}$) was significantly increased by STZ-DM under all conditions, although not in mice treated with high doses of MTF (Figure 2C). SM metabolic response to diabetes was, at least partially, confirmed by the conventional analysis of FDG uptake indexed by SUV that was significantly higher in the SM of untreated STZ-DM mice with respect to corresponding controls (Figure 2D, E). By contrast, there was virtually no appreciable MTF effect in any of the studied conditions (Figure 2D, E).

### 3.3 Effect of diabetes and MTF on enzymatic pathways in SM

**Figure 3:**
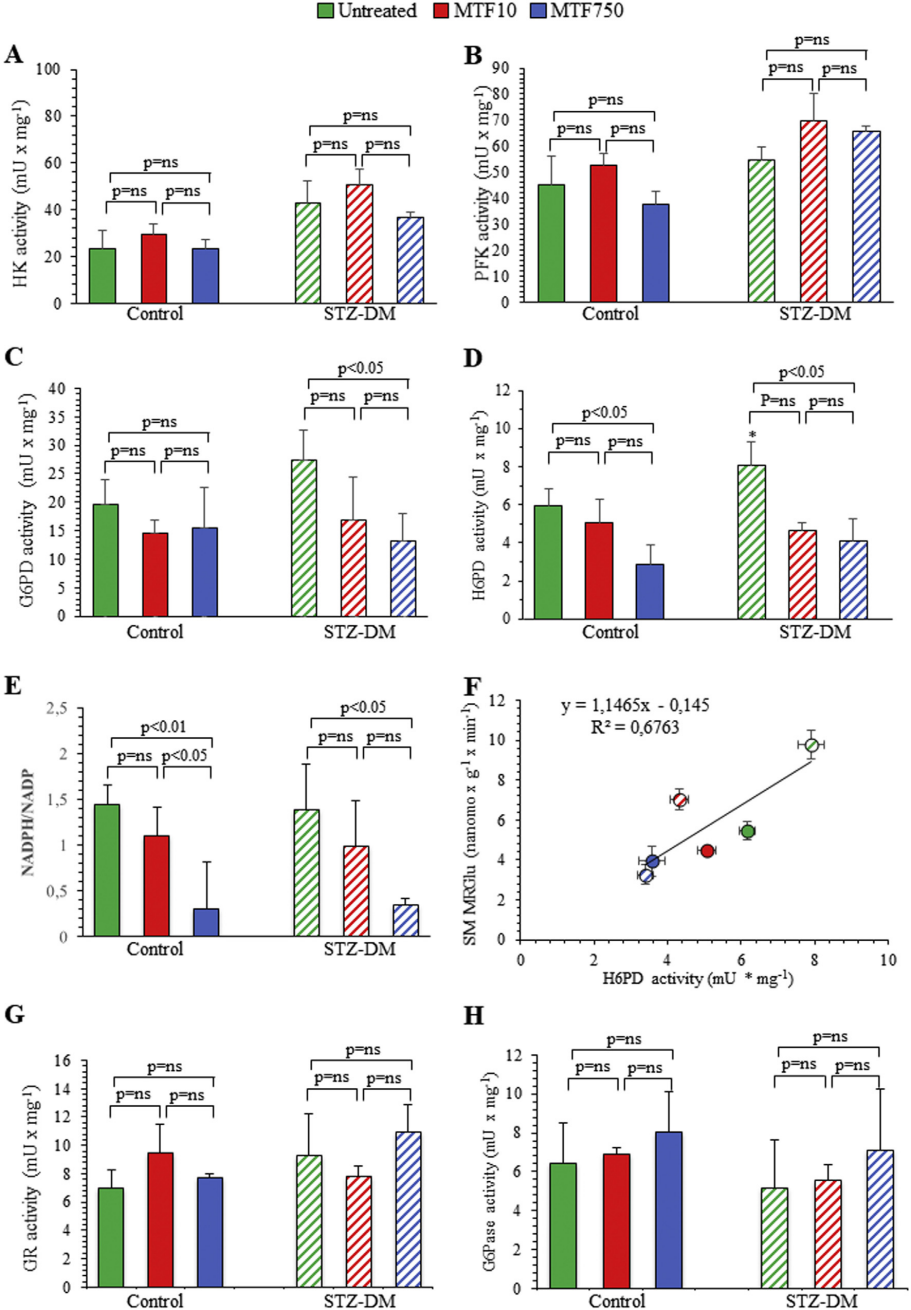
*Effect of diabetes and metformin on the enzymatic pathway.* Catalytic activities of HK (A), PFK (B), G6PD (C), and H6PD (D) in control (solid columns) and STZ-DM groups (dashed columns) of untreated (green) or treated with low-dose (red) or high-dose (blue) MTF (right) SM homogenate. The NADPH/NADP ratio is represented in panel E. The correlation between H6PD activity and MRGlu (F). GR and G6Pase catalytic activities are expressed in panels G and H, respectively. *p < 0.05 vs the corresponding group of nondiabetic mice.

The effect of STZ-DM and MTF on SM avidity for FDG was not explained by the activity of enzymes governing cytosolic glucose catabolism. The catalytic function of HK was increased by STZ-DM, although it was virtually not affected by MTF in either group (Figure 3A). A comparable trend was also evident for PFK (Figure 3B). Similarly, G6PD at least partially mimicked the response of HK and PFK: its activity was substantially similar in all nondiabetic groups, while the slight (and not significant) increase induced by STZ-DM was inhibited only by high-dose MTF (Figure 3C).

Accordingly, the analysis of the regulators of cytosolic pathways of glucose degradation could not explain the observed response of either MRGlu or avidity for FDG to both STZ-DM and MTF. We thus moved our attention to the ER enzyme H6PD: actually, its catalytic function was slightly increased in SM harvested from STZ-DM mice as compared with controls (Figure 3D). On the other hand, it was significantly reduced by MTF-750 regardless of the presence or absence of diabetes. Similarly, the NADPH/NADP ratio was increased in the presence of hyperglycemia, while it was decreased by MTF treatment (Figure 3E), nicely duplicating the response of SM in terms of H6PD function and avidity for FDG.

This observation was corroborated by the evidence of a direct linear relationship between H6PD function and corresponding MRGlu in SM harvested from both CTR and STZ-DM mice (Figure 3F). Finally, glutathione-reductase activity was slightly, though not significantly, enhanced by chronic hyperglycemia, while it was not significantly affected by MTF in either control or STZ-DM mice (Figure 3G). By contrast, the activity of G6Pase was not significantly influenced by either STZ-DM or MTF (Figure 3H).

### 3.4 Ex vivo glucose avidity and FDG accumulation kinetics

**Figure 4:**
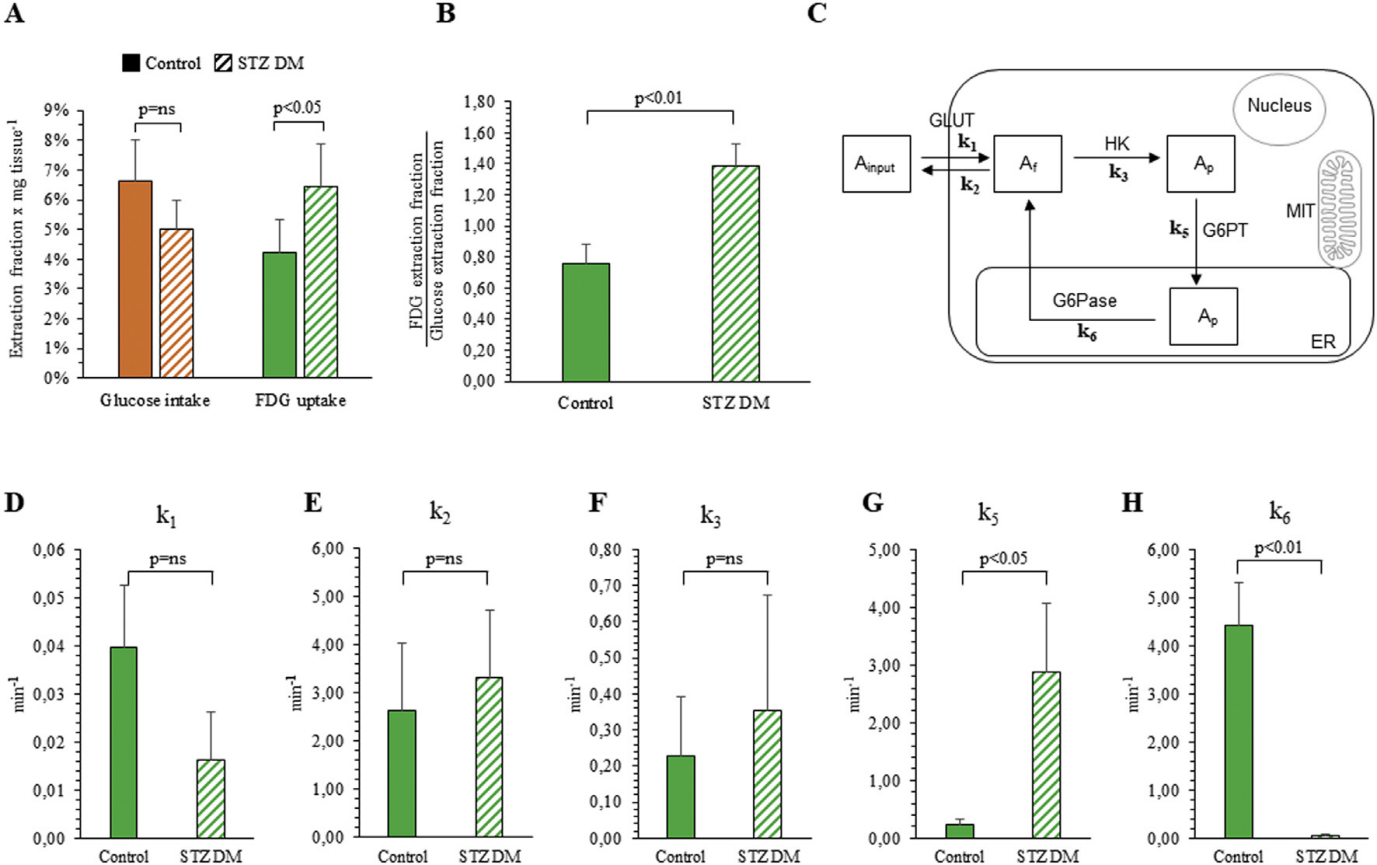
*Ex vivo glucose intake and FDG uptake kinetics.* Glucose consumption (orange) and FDG fractional uptake (green) in control (solid columns) and STZ-DM of harvested SM (dashed columns) (n = 3, A). Panel B displays the in vitro lumped constant (the ratio between the extraction fractions of FDG and glucose). Panel C displays the proposed 4-C model to describe the ER role in accumulation and loss of FDG. Panels D–H show values in rate constants.

The disagreement between glucose intake and FDG uptake was confirmed by the ex vivo analysis. In fact, over the 45-min experiment, SM avidity for the tracer showed divergent responses to STZ-DM: glucose consumption slightly (though not significantly) decreased. By contrast, FDG fractional uptake increased by almost 50% (Figure 4A). As a consequence, the in vitro lumped constant (the ratio between the extraction fractions of FDG and glucose) was almost twofold in STZ-DM as compared with control SM (1.39 ± 0.14 vs 0.76 ± 0.12, respectively, *p* < 0.01, Figure 4B).

As shown in Figure 4, the difference in tracer avidity was not explained by divergent values in rate constants of transmembrane exchanges (k_1_), which were even lower in hyperglycemic mice. Similarly, FDG back-transfer to supernatant (k_2_) remained constant (Figure 4D, E). Moreover, the increased accumulation rate was not associated with a higher rate of phosphorylation catalysis, since k_3_ was not significantly different between the two experimental conditions (Figure 4F). By contrast, it was associated with a marked (>10-fold) increase in FDG-6P transport to the ER (k_5_) (Figure 4G), combined with an even more obvious reduction (nearly 100-fold) in dephosphorylation rate and back flux to the cytosol (k_6_) (Figure 4H).

### 3.5 Immunofluorescence analysis

**Figure 5:**
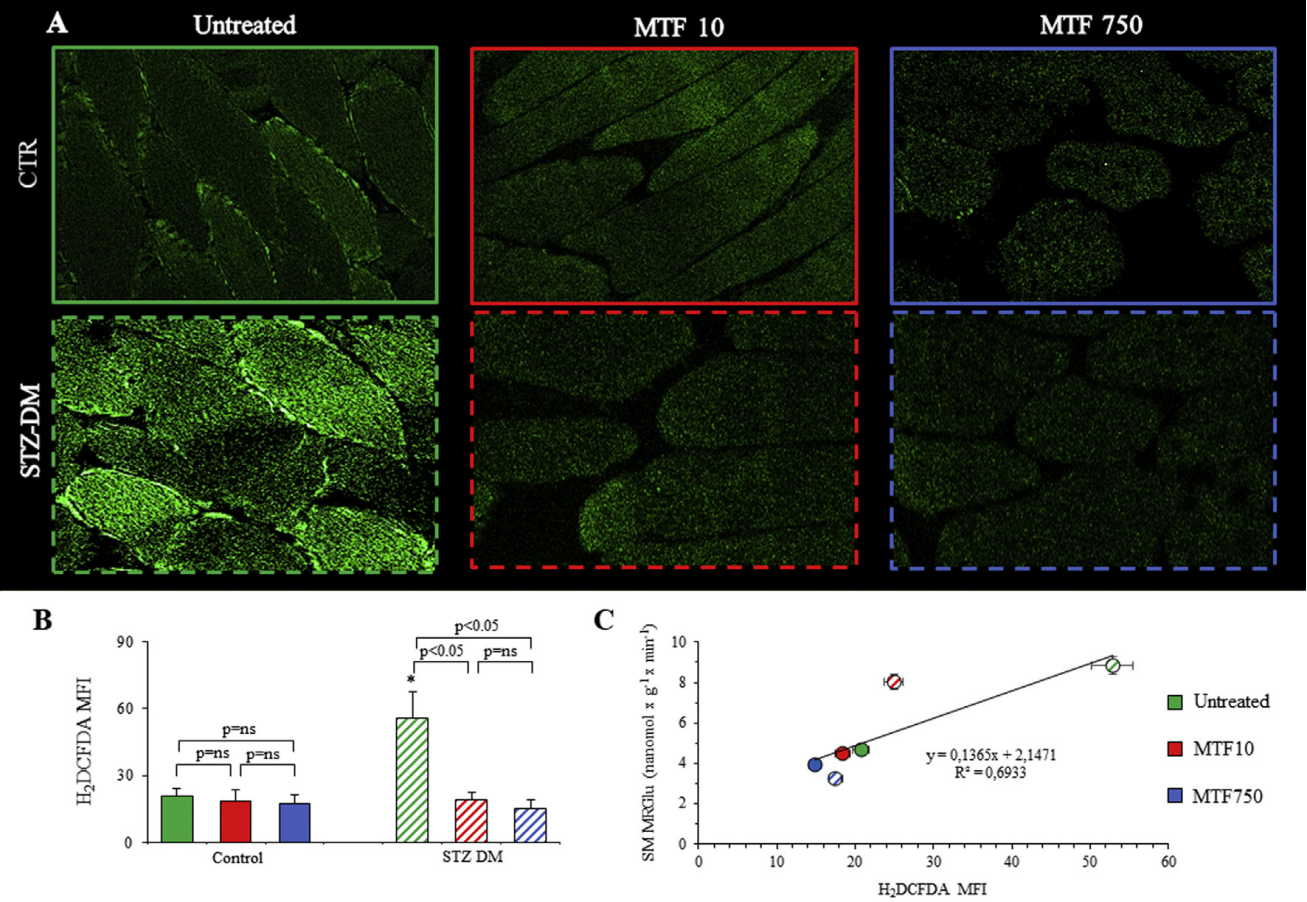
*Muscle sections in the redox state (ROS production).* Representative images of muscle sections from control (CTRL) and STZ-treated mice (STZ-DM) stained with H_2_DCFDA (A) to assess the presence of ROS. Mean fluorescence index (MFI) of H_2_DCFDA (B) of CTRL and STZ-DM mice untreated (green) or under low-dose (red) or high-dose (blue) MTF. Correlation between H_2_DCFDA (*x*-axis) and MRGlu (*y*-axis) (C). n = 3; *p < 0.05 vs the corresponding group of nondiabetic mice.

**Figure 6:**
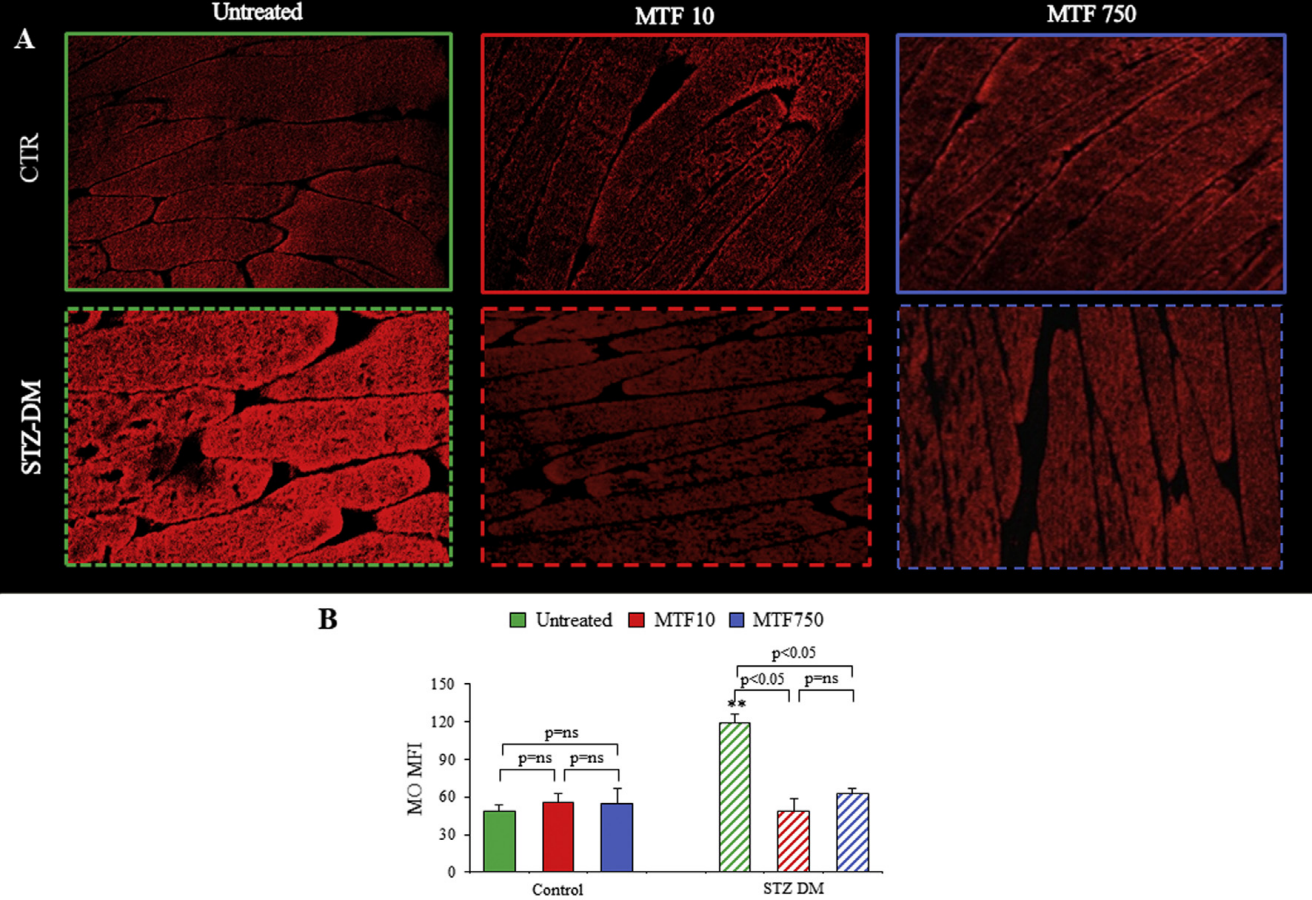
*Muscle sections in the redox state (antioxidant response).* Representative images of muscle sections from control (CTRL) and STZ-treated mice (STZ-DM) stained with Mercury Orange (A) to assess the presence of free thiols. The mean fluorescence index (MFI) of Mercury Orange (B) of the CTRL and STZ-DM mice untreated (green) or under low-dose (red) or high-dose (blue) of MTF. n = 3; **p < 0.01 vs the corresponding group of nondiabetic mice.

Among the series of signals regulating PPP activity, the need to feed NADPH to antioxidant responses plays a significant contribution. Accordingly, we evaluated the indexes of both oxidative stress and antioxidant responses in SM sections harvested from control or STZ-DM mice. Staining with H_2_DCFDA documented a significant fluorescence increase, and thus a greater ROS generation, in SM harvested from STZ-DM mice as compared with control ones (Figure 5). A similar conclusion was provided by the analysis of MO staining that reported a significant increase in glutathione-dependent antioxidant response in SM exposed to chronic hyperglycemia (Figure 6). The increase in H_2_DCFDA and MO fluorescence induced by STZ-DM was prevented by both MTF doses that reported fluorescence intensity to values superimposable to the control ones. Moreover, the link between FDG uptake and ROS generation was confirmed by the presence of a direct correlation between the mean fluorescence index of H_2_DCFDA and SM MRGlu (Figure 5C), although this relationship was not reproduced by the index of antioxidant response provided by MO (data not shown).

## 4 Discussion

FDG uptake was increased in hindlimb SM of hyperglycemic mice under fasting state and thus in the absence of insulin stimulation. This metabolic feature reflected an increased hexose avidity, documented by an accelerated rate constant of FDG accumulation despite the competition by high serum levels of unlabeled glucose. This response was independent of either catalytic function of enzymes controlling cytosolic glucose degradation. By contrast, it closely matched the response of H6PD, whose activity was enhanced by STZ-DM through a mechanism that was inhibited by MTF. The role of this reticular pathway was confirmed by ex vivo compartmental analysis that indicated a selective enhancement of FDG accumulation rate within the ER of SM harvested from STZ-DM mice. Finally, the evident response of H6PD, and the consequent activation of ER PPP, were coherent with the activation of NADPH-dependent antioxidant responses to the increase in ROS generation caused by chronic hyperglycemia.

The accelerated FDG uptake and the consequent high value of estimated MRGlu in SM of chronically hyperglycemic mice apparently conflict with a large body of literature in type 2 diabetes. However, most of these studies used the so-called clamp maneuver to estimate the impairment in FDG transport or phosphorylation under steadily elevated serum insulin stimulation. In the present experiments, imaging was performed after 12 h of fasting, to decrease insulin concentration to its minimal value. Under this condition, published data about FDG uptake in SM are relatively scarce and largely more uncertain. Kelley et al. observed a slight reduction in SM MRGlu in subjects with type 2 diabetes [38]. However, this finding has not been confirmed by several authors (including the same group), who reported similar values of nonstimulated SM MRGlu in fasting diabetic and normal subjects [39,40], considering hyperglycemia as a compensatory response counteracting diabetes-related impairment in glucose intake [[41], [42], [43]].

Together with the direct association between extensive muscle FDG uptake and high serum glucose levels reported in a large clinical series of cancer patients [44], these considerations indicate a modest (and variable) interference of hyperglycemia on FDG uptake in fasting SM. Yet this conclusion strikingly conflicts with the direct measurement of glucose intake, since the estimation of leg balance of ^3^H-3glucose using the Fick principle showed an almost halving of MRGlu in hyperglycemic patients with type 2 diabetes [45]. The strict link between H6PD activity and SM uptake of FDG provides a first partial explanation for this disagreement. Actually, it does not fit with the classical three-compartment model for interpreting FDG uptake. Nevertheless, it agrees with the notion that almost one-half of FDG taken up by the cell is transformed in metabolites downstream from FDG6P through pathways only partially shared with glucose [[46], [47], [48], [49]]. On the other hand, a low, yet measurable, radioactivity loss has been observed in virtually all studied tissues after uptake of either ^14^C-2DG or FDG. This washout has been indisputably attributed to the hydrolysis of phosphorylated hexoses catalyzed by G6Pase. A large body of literature more recently documented that this enzyme is confined within the ER lumen [50,51], implicitly asking for a transport mechanism able to carry the polar hexose through the ER membrane to the G6Pase catalytic site. In agreement with this consideration, FDG radioactivity loss has been found to reflect the expression of the unidirectional ATP-dependent transporter G6PT much more than the activity of G6Pase [52].

These considerations thus indicate that FDG6P is indeed conveyed into the ER. This concept is corroborated by the ex vivo experiments whose capability to maintain steady-state concentrations of tracer (FDG) and tracee (glucose) permitted the optimization of compartmental analysis of FDG kinetics in SM. This approach indeed documented a mismatch in the response to STZ-DM that selectively increased tracer uptake while decreasing directly estimated MRGlu in a sample devoid of the interference by competing metabolites and signaling hormones. Complementing our previous observations [[10], [11], [12], [13]], the selective increase in FDG extraction fraction induced by STZ-DM was not associated with significant changes in the recognized determinants of the lumped constant, that is, transmembrane transport (k_1_ and k_2_) and phosphorylation (k_3_). By contrast, it reflected an acceleration in radioactivity transfer from the cytosol to the ER (k_5_), combined with an even more evident deceleration of its reverse backflow (k_6_) (Figure 4). Intriguingly, this mathematical analysis agreed with the increase in H6PD activity, while it was not associated with changes in G6Pase catalytic function. This observation suggests a direct competition between these two enzymes within the ER lumen. According to this model, any functional shift in the activity of each player modifies the availability of FDG6P for the counterpart as to accelerate G6Pase-mediated radioactivity release or H6PD-facilitated accumulation [30].

**Figure 7:**
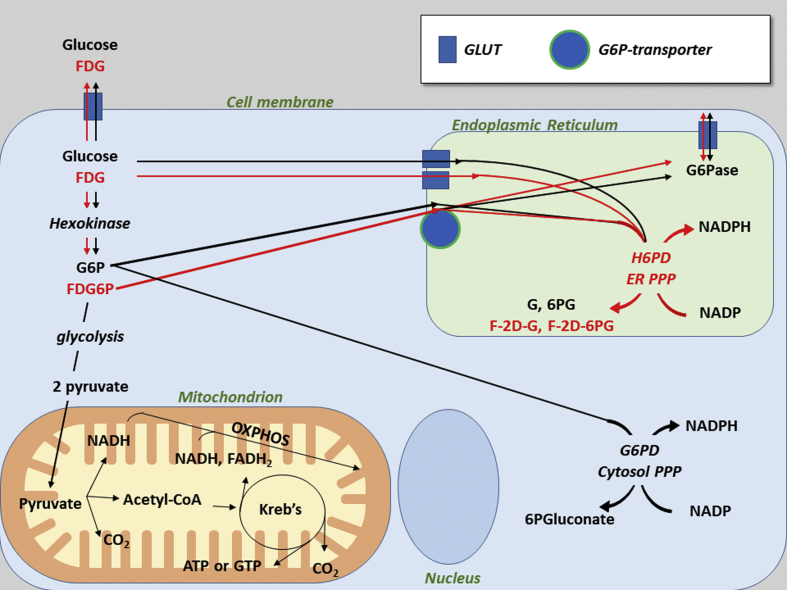
*Proposed model of SM-FDG uptake and its relationship with NADP reduction to NADPH.* The cartoon proposes the ER contribution to FDG kinetics. Pathways of glucose and FDG are depicted in black and red, respectively. G, gluconate; 6PG, 6-P-gluconate; F-2D-G, fluoro-deoxy-gluconate; F-2D-PG, fluoro-deoxy-6P-gluconate. Gluts are depicted as squares, G6P-transporter as a circle.

The present data do not clarify whether the reticular fate is a selective feature of FDG and FDG6P or is shared by other hexoses. However, the ER covers a double role (target and contributor) in cell redox control, while glucose metabolism is strictly interconnected with all the canonical branches of unfolded protein response [53]. In agreement with this concept, the abundance of transport mechanisms acting on free and phosphorylated hexoses in the reticular membrane [54] is complemented by the presence of a full PPP local enzymatic asset [55]. One of the main roles of PPP is the maintenance of NADPH levels needed for bioreductive syntheses or glutathione-dependent antioxidant responses. In agreement with previous studies [56,57], SM harvested from chronically hyperglycemic mice showed an enhanced ROS generation counterbalanced by an activated antioxidant response, while the STZ-DM–related redox stress was significantly prevented by both MTF doses. Interestingly, the response of these histochemical markers to both hyperglycemia and biguanide nicely fitted the behavior of H6PD and FDG uptake. In other words, the response of radioactivity retention to STZ-DM was coherent with the expected increase in NADPH utilization for the ER response to the oxidative stress associated with chronic hyperglycemia [15,16]. Similarly, the selective effect of MTF in STZ-DM fits the expected decrease in ROS generation caused by this drug [58] either directly (through the biguanide-related respiratory inhibition) or indirectly (through the drug's antihyperglycemic action). Should these data be confirmed, they would indicate a pivotal role of H6PD, besides the most acknowledged G6PD, in the control of overall cell NADPH/NADP ratio (Figure 7).

## 5 Conclusion

The present data indicate that fasting FDG uptake at least partially reflects H6PD activity and is thus enhanced under the redox stress induced by hyperglycemia despite the competition of serum concentration of unlabeled glucose. From the methodological point of view, the selected model and the absence of insulin assay do not permit us to extend the contribution of this reticular metabolism to FDG accumulation under insulin-stimulated conditions in diabetic patients [40]. From the biochemical perspective, these findings indicate that the cell metabolic response to hyperglycemia-induced redox stress involves the activation of H6PD-triggered PPP and thus a selective modulation of the NAPDH/NADP ratio within the ER.

## Ethics approval

All procedures involving animals were performed in accordance with the current national and international regulations and were reviewed and approved by the Licensing and Animal Welfare Body of the IRCCS Policlinico San Martino, Genoa, Italy, and by the Italian Ministry of Health.

## Funding

This study was funded by the program “Ricerca Corrente,” line “Guest–Cancer Interactions,” by Compagnia di San Paolo (project ID Prot.: 2015.AAI4110.U4917).

## Conflict of interests

None declared.
