# Supplementary material for: FDG uptake tracks the oxidative damage in diabetic skeletal muscle: An experimental study
Source: Mol Metab. 2019 Nov 15;31:98–108. doi: 10.1016/j.molmet.2019.11.007 (PMC6920267; doi:10.1016/j.molmet.2019.11.007)
Supplement: Multimedia component 1 [file mmc1.pptx]

## Slide 1
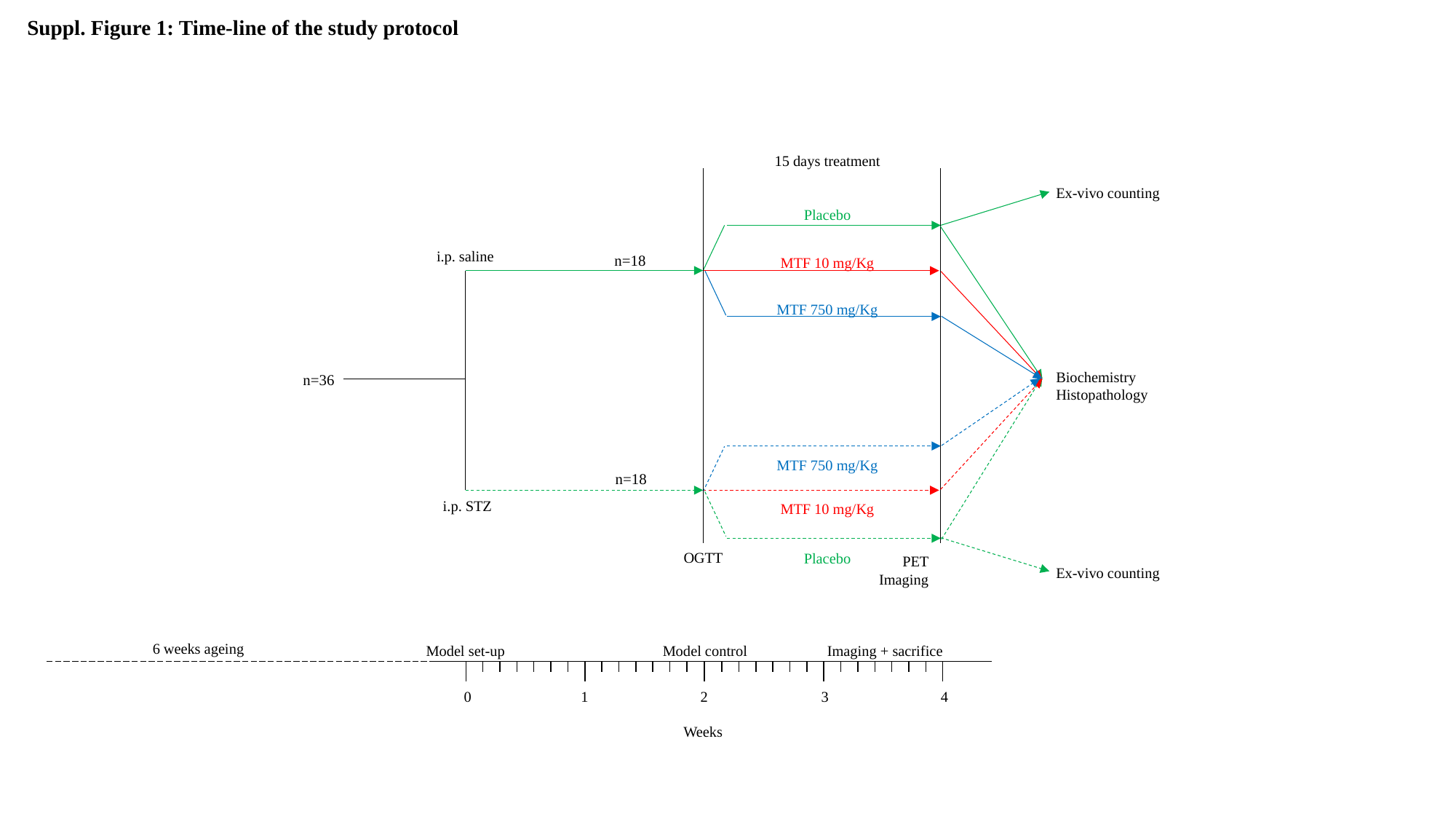

Suppl. Figure 1: Time-line of the study protocol
15 days treatment
Placebo
Ex-vivo counting
i.p. saline
MTF 10 mg/Kg
n=18
MTF 750 mg/Kg
Biochemistry
Histopathology
n=36
MTF 750 mg/Kg
n=18
MTF 10 mg/Kg
i.p. STZ
Placebo
Ex-vivo counting
OGTT
PET
Imaging
6 weeks ageing
Model set-up
Model control
Imaging + sacrifice
0
1
2
3
4
Weeks
